# Social Inequalities in Young Children’s Meal Skipping Behaviors: The Generation R Study

**DOI:** 10.1371/journal.pone.0134487

**Published:** 2015-07-30

**Authors:** Anne I. Wijtzes, Wilma Jansen, Vincent W. V. Jaddoe, Oscar H. Franco, Albert Hofman, Frank J. van Lenthe, Hein Raat

**Affiliations:** 1 The Generation R Study Group, Erasmus Medical Center, Rotterdam, The Netherlands; 2 Department of Public Health, Erasmus Medical Center, Rotterdam, The Netherlands; 3 Department of Social Development, City of Rotterdam, Rotterdam, The Netherlands; 4 Department of Pediatrics, Erasmus Medical Center, Rotterdam, The Netherlands; 5 Department of Epidemiology, Erasmus Medical Center, Rotterdam, The Netherlands; Institute of Preventive Medicine, DENMARK

## Abstract

**Background:**

Regular meal consumption is considered an important aspect of a healthy diet. While ample evidence shows social inequalities in breakfast skipping among adolescents, little is known about social inequalities in breakfast skipping and skipping of other meals among young school-aged children. Such information is crucial in targeting interventions aimed to promote a healthy diet in children.

**Methods:**

We examined data from 4704 ethnically diverse children participating in the Generation R Study, a population-based prospective cohort study in Rotterdam, the Netherlands. Information on family socioeconomic position (SEP), ethnic background, and meal skipping behaviors was assessed by parent-reported questionnaire when the child was 6 years old. Multiple logistic regression analyses were performed to assess the associations of family SEP (educational level, household income, employment status, family composition) and ethnic background with meal skipping behaviors, using high SEP children and native Dutch children as reference groups.

**Results:**

Meal skipping prevalence ranged from 3% (dinner) to 11% (lunch). The prevalence of meal skipping was higher among low SEP children and ethnic minority children. Maternal educational level was independently associated with breakfast skipping ([low maternal educational level] OR: 2.21; 95% CI: 1.24,3.94). Paternal educational level was independently associated with lunch skipping ([low paternal educational level] OR: 1.53; 95% CI: 1.06,2.20) and dinner skipping ([mid-high paternal educational level] OR: 0.39; 95% CI: 0.20,0.76). Household income was independently associated with breakfast skipping ([low income] OR: 2.43, 95% CI: 1.40,4.22) and dinner skipping ([low income] OR: 2.44; 95% CI: 1.22,4.91). In general, ethnic minority children were more likely to skip breakfast, lunch, and dinner compared with native Dutch children. Adjustment for family SEP attenuated the associations of ethnic minority background with meal skipping behaviors considerably.

**Conclusion:**

Low SEP children and ethnic minority children are at an increased risk of breakfast, lunch, and dinner skipping compared with high SEP children and native Dutch children, respectively. Given these inequalities, interventions aimed to promote regular meal consumption, breakfast consumption in particular, should target children from low socioeconomic groups and ethnic minority children. More qualitative research to investigate the pathways underlying social inequalities in children’s meal skipping behaviors is warranted.

## Introduction

Healthy dietary behaviors are important determinants of children’s development and health outcomes, including nutritional status, weight status, and cognition [1–3]. One element of a healthy diet is regular meal consumption [4], of which breakfast consumption has been studied most extensively [2, 3, 5]. Regular breakfast consumption has been associated with overall diet quality (e.g. more servings of fruits, vegetables, grains, and dairy products, and less consumption of unhealthy snacks and soft drinks), lower body mass index (BMI), and increased cognitive function and academic performance [1–3, 5–10]. Consequentially, daily consumption of a nutrient-dense breakfast has been included in the dietary guidelines for Americans as published by the US departments of Agriculture and Health and Human Services [11].

Identification and characterization of children at high risk of breakfast skipping is a crucial step in the process of designing interventions aimed to promote daily breakfast consumption. Previous research has shown that adolescents with an ethnic minority background and adolescents from a family with a low socioeconomic position (SEP) are more likely to skip breakfast compared with their counterparts [12–17]. Furthermore, adolescents from single parent families are more likely to skip breakfast than adolescents from two-parent families [18–20]. Studies on the associations of family SEP and ethnic background with breakfast skipping in young school-aged children are scarce [6, 9], as are studies on social determinants of children’s lunch and dinner skipping in general [17]. Given that dietary behaviors track through childhood and into adolescence [21, 22], identification of risk groups at an early age is warranted. Furthermore, despite ample evidence on the associations between breakfast skipping and diet quality, little is known on the extent to which meal skipping behaviors co-occur.

Therefore, the aim of this study was twofold. First, we aimed to assess the prevalence and co-occurrence of breakfast skipping, lunch skipping, and dinner skipping in 6-year-old children. Second, we aimed to assess the associations of family SEP (as indicated by parental educational level, parental employment status, household income, and family composition) and ethnic background with these meal skipping behaviors. The present study used data from the Generation R Study, a large multi-ethnic birth cohort study in Rotterdam, the Netherlands.

## Methods

### Study design

This study was embedded in the Generation R Study, a population-based prospective cohort study from fetal life onwards. The Generation R Study was designed to identify early environmental and genetic determinants of growth, development, and health, and has been described previously in detail [23]. The study was conducted in accordance with the guidelines proposed in the World Medical Association Declaration of Helsinki and has been approved by the Medical Ethical Committee at Erasmus MC, University Medical Center Rotterdam. Written informed consent was obtained from all parents.

### Study population

Invitations to participate in the study were made to all pregnant women who had an expected delivery date between April 2002 and January 2006 and who lived in the study area (Rotterdam, the Netherlands) at time of delivery. From the original 9749 known live born children of the Generation R cohort, 8305 children still participate in the school-aged period (5 years onward) [23]. For this study, we selected children born to mothers with a native Dutch, Surinamese-Creole, Surinamese-Hindustani, Dutch Antillean, Cape Verdean, Turkish, or Moroccan ethnic background (n = 6447). These ethnic groups were chosen because they represent the largest ethnic groups in the Generation R Study, as well as in the city of Rotterdam [23]. Children with missing data on all three meal skipping behaviors were excluded (n = 1347). To avoid clustering of data, we furthermore excluded second (n = 389) and third children (n = 7) of the same mother, leaving a study population of 4704 participants. Of these participants, 4687 had information on breakfast skipping, 4593 had information on lunch skipping, and 4537 had information on dinner skipping.

### Meal skipping behaviors

Meal skipping behaviors were assessed in parent-reported questionnaires at child age 6 years. Parents were asked to think of an average week when reporting their children’s meal skipping behaviors. Number of days on which children consumed breakfast, lunch, and dinner was assessed for weekdays (6 answer options ranging from 0 to 5 days) and weekend days separately (3 answer options ranging from 0 to 2 days), and these were added to calculate weekly consumption (8 answer options ranging from 0 to 7 days) (S1 Table). Based on a highly skewed distribution of the data, as well as previously used definitions of meal skipping among children and adolescents [6, 7, 12, 14, 19], skipping a meal was defined as consumption less than 7 days per week.

### Family socioeconomic position and ethnic background

Information on family SEP and ethnic background was assessed by parent-reported questionnaire when the child was 6 years old. Indicators of family SEP included maternal and paternal educational level (highest level attained), maternal and paternal employment status (no paid job, paid job), and net household income (<€2000/month [i.e. below modal income [24]], €2000-€3200/month, >€3200/month). The Dutch Standard Classification of Education was used to categorize four levels of education: low (no education, primary school, lower vocational training, intermediate general school, or three years or less general secondary school), mid-low (more than three years general secondary school, intermediate vocational training, or first year of higher vocational training), mid-high (higher vocational training), and high (university or PhD degree) [25]. Albeit not a traditional SEP indicator [26], we also included family composition (single parent, two parents [not necessarily biological parents]) as indicator of family SEP as it has been used as a proxy indicator of SEP in previous research and has been consistently associated with breakfast skipping [18–20, 27]. Children’s ethnic background was based on the ethnic background of their mothers to take into account the cultural background of the mothers (most often primary caregivers). Maternal ethnic background was based on country of birth of the mother’s parents. In accordance with Statistics Netherlands, a mother was considered nonnative Dutch if one of her parents was born abroad. If both parents were born abroad, country of birth of the mother’s mother decided on maternal ethnic background [28].

### Potential confounders

Child’s sex and age were considered potential confounders in the associations of family SEP and ethnic background with children’s meal skipping behaviors. When assessing the associations of family SEP with children’s meal skipping behaviors, ethnic background was considered a potential confounder, and vice versa.

### Statistical analyses

Descriptive statistics were used to characterize the study population. Meal skipping behaviors according to family SEP and ethnic background were assessed using Chi-square tests. Spearman’s rho correlation coefficients were calculated to assess the correlation between children’s relative rank positions in number of days of breakfast, lunch, and dinner skipping (0–7 days, S1 Table). Furthermore, cross-tabulations of the dichotomized meal skipping variables (yes, no) were used to assess the proportion of lunch and dinner skippers among breakfast skippers and the proportion of lunch and dinner consumers among breakfast consumers.

Associations of family SEP with meal skipping behaviors at age 6 years were assessed using series of multiple logistic regression analyses with high SEP children as the reference group. First, we created crude models (i.e. unadjusted models) and basic models adjusted for confounders (i.e. child’s sex, age at measurement, and ethnic background). To assess the independent effects of each of the SEP indicators, full models additionally contained all SEP indicators simultaneously. For ethnic background, similar logistic regression models were built using native Dutch children as the reference group. First, crude models and basic models adjusted for basic confounders (i.e. child’s sex and age at measurement) were built. To adjust for confounding effects by family SEP, full models were additionally adjusted for all indicators of family SEP. Collinearity analysis using Spearman’s rho coefficients yielded acceptable collinearity (r<0.8) between maternal educational level, paternal educational level, and household income; therefore, these variables were included simultaneously in the full models. To assess differences between ethnic minority groups, additional analyses were performed using the group with the highest risk of meal skipping as the reference group. Furthermore, additional analyses on socioeconomic and ethnic inequalities in weekly consumption of meals (0–21) were performed (S2 and S3 Tables).

A multiple imputation procedure was applied to handle missing data in the family SEP variables [29]. Five imputed datasets were generated using a fully conditional specified model, thus taking into account the uncertainty of the imputed values. Pooled estimates from these five imputed datasets were used to report beta’s, odds ratios (ORs) and their 95% confidence intervals (CIs). Imputations were based on the relationships between all the variables included in this study. All analyses were conducted with Statistical Package for Social Sciences (SPSS) version 21.0 for Windows (IBM Corp., Armonk, NY, USA). A significance level of p<0.05 was used to indicate significant associations.

### Nonresponse analyses

Children with missing data on all three meal skipping behaviors (n = 1347) were compared to children with information on at least one behavior (n = 5100) using Chi-square tests. Data were more often missing for ethnic minority children (χ2 = 473, df = 6, p<0.001), children with a low maternal educational level (χ2 = 25, df = 3, p<0.001), and children with a low household income (χ2 = 15, df = 2, p<0.01). Nonresponse did not differ according to any of the other socioeconomic indicators (all p>0.05).

## Results

Table 1 shows characteristics of the study population. The majority of children had a Dutch ethnic background (70.7%). Approximately half of the children had a mother with a low or mid-low educational level (45.6%). The prevalence of meal skipping in the total study population was 6.4%, 10.6%, and 3.1% for breakfast, lunch, and dinner skipping, respectively. Meal skipping behaviors were more prevalent among children with an ethnic minority background and children from low SEP families, irrespective of SEP indicator (Table 2). Spearman’s rho correlation coefficients were 0.26 for breakfast and lunch skipping, 0.36 for breakfast and dinner skipping, and 0.31 for lunch and dinner skipping (all p<0.001). Of those children who skipped breakfast, 41% also skipped lunch, and 28% also skipped dinner. Of those children who consumed breakfast, 92% also consumed lunch, and 99% also consumed dinner.

**Table 1:** Characteristics of the study population (n = 4704).

		Total	Missing
		n (%)	n (%)
*Social characteristics*			
Maternal educational level	High	1240 (26.8)	67 (1.4)
	Mid-high	1280 (27.6)	
	Mid-low	1480 (31.9)	
	Low	637 (13.7)	
Paternal educational level	High	1387 (32.9)	489 (10.4)
	Mid-high	964 (22.9)	
	Mid-low	1134 (26.9)	
	Low	730 (17.3)	
Maternal employment status	Paid job	3324 (75.6)	308 (6.5)
	No paid job	1072 (24.4)	
Paternal employment status	Paid job	3894 (94.1)	566 (12.0)
	No paid job	244 (5.9)	
Household income	>€3200	2169 (49.6)	336 (7.1)
	€2000-<€3200	1144 (26.2)	
	<€2000	1055 (24.2)	
Family composition	Two parents	4002 (85.8)	37 (0.8)
	Single parent	665 (14.2)	
Ethnic background	Native Dutch	3324 (70.7)	0
	Surinamese-Creole	161 (3.4)	
	Surinamese-Hindustani	168 (3.6)	
	Dutch Antillean	124 (2.6)	
	Cape Verdean	193 (4.1)	
	Turkish	458 (9.7)	
	Moroccan	276 (5.9)	
*Child characteristics*			
Sex	Boy	2380 (50.6)	0
	Girls	2324 (49.4)	
Age	Months (90% range)	71.6 (67.7–84.5)	0
Breakfast skipping	No	4389 (93.6)	17 (0.4)
	Yes	298 (6.4)	
Lunch skipping	No	4107 (89.4)	111 (2.4)
	Yes	486 (10.6)	
Dinner skipping	No	4395 (96.9)	
	Yes	142 (3.1)	167 (3.6)

Table is based on non-imputed dataset.

**Table 2:** Meal skipping behaviors according to family socioeconomic position and ethnic background (n = 4704).

		Breakfast skipping		p-Value*	Lunch skipping		p-Value*	Dinner skipping		p-Value*
		(n = 4687)			(n = 4593)			(n = 4537)		
		No	Yes		No	Yes		No	Yes	
		n (%)	n (%)		n (%)	n (%)		n (%)	n (%)	
Maternal educational level	High	1214 (97.9)	26 (2.1)	<0.001	1128 (92.8)	88 (7.2)	<0.001	1185 (98.2)	22 (1.8)	<0.01
	Mid-high	1222 (95.5)	57 (4.5)		1139 (90.8)	115 (9.2)		1205 (97.2)	35 (2.8)	
	Mid-low	1363 (92.6)	109 (7.4)		1277 (88.6)	165 (11.4)		1376 (96.8)	46 (3.2)	
	Low	537 (85.1)	94 (14.9)		512 (82.8)	106 (17.2)		576 (94.7)	32 (5.3)	
Paternal educational level	High	1344 (96.9)	43 (3.1)	<0.001	1259 (92.8)	98 (7.2)	<0.001	1311 (97.5)	34 (2.5)	<0.01
	Mid-high	928 (96.6)	33 (3.4)		882 (92.5)	72 (7.5)		929 (98.6)	13 (1.4)	
	Mid-low	1052 (93.2)	77 (6.8)		965 (87.8)	134 (12.2)		1054 (96.8)	35 (3.2)	
	Low	637 (88.0)	87 (12.0)		598 (84.6)	109 (15.4)		665 (95.3)	33 (4.7)	
Maternal employment status	Paid job	3158 (95.2)	159 (4.8)	<0.001	2948 (90.6)	306 (9.4)	<0.001	3136 (97.4)	84 (2.6)	<0.01
	No paid job	964 (90.3)	103 (9.7)		902 (86.8)	137 (13.2)		980 (95.8)	43 (4.2)	
Paternal employment status	Paid job	3684 (94.9)	200 (5.1)	<0.001	3447 (90.5)	363 (9.5)	<0.05	3672 (97.5)	95 (2.5)	<0.001
	No paid job	204 (84.6)	37 (15.4)		201 (85.9)	33 (14.1)		218 (92.4)	18 (17.6)	
Household income	>€3200	2118 (97.7)	50 (2.3)	<0.001	1966 (92.2)	166 (7.8)	<0.001	2074 (98.3)	36 (1.7)	<0.001
	€2000-<€3200	1062 (92.9)	81 (7.1)		993 (88.8)	125 (11.2)		1070 (96.7)	36 (3.3)	
	<€2000	902 (86.4)	142 (13.6)		850 (83.7)	165 (16.3)		940 (94.2)	58 (5.8)	
Family composition	Two parents	3767 (94.3)	226 (5.7)	<0.001	3528 (90.1)	388 (9.9)	<0.001	3763 (97.2)	110 (2.8)	<0.01
	Single parent	586 (89.2)	71 (10.8)		548 (85.4)	94 (14.6)		598 (95.2)	30 (4.8)	
Ethnic background	Native Dutch	3324 (97.1)	95 (2.9)	<0.001	2988 (91.7)	272 (8.3)	<0.001	3159 (98.0)	64 (2.0)	<0.001
	Surinamese-Creole	139 (86.3)	22 (13.7)		133 (85.8)	22 (14.2)		142 (92.8)	11 (7.2)	
	Surinamese-Hindustani	147 (88.0)	20 (12.0)		153 (92.7)	12 (7.3)		153 (95.0)	8 (5.0)	
	Dutch Antillean	108 (87.8)	15 (12.2)		106 (86.9)	16 (13.1)		117 (95.1)	6 (4.9)	
	Cape Verdean	173 (90.6)	18 (9.4)		156 (83.9)	30 (16.1)		171 (93.4)	12 (6.6)	
	Turkish	372 (82.3)	80 (17.7)		338 (77.9)	96 (13.1)		409 (94.7)	23 (5.3)	
	Moroccan	226 (82.5)	48 (17.5)		233 (86.0)	38 (14.0)		244 (93.1)	18 (6.9)	

Table is based on non-imputed dataset.

* P-Values assessed by Chi-square tests

Tables 3–5 show the associations of family SEP and ethnic background with children’s breakfast, lunch, and dinner skipping. After adjustment for child’s sex, age, and ethnic background, a low maternal educational level, a low paternal educational level, a low household income, and a single parent family were associated with an increased risk of breakfast skipping (basic model, Table 3). Similar associations were found for the associations between family SEP and lunch skipping (Table 4). Results were slightly different for dinner skipping, where associations were found with a low maternal educational level, mid-high paternal educational level, paternal employment status (no paid job) and a low household income (Table 5). After adjustment for other SEP indicators, maternal educational level and household income remained associated with children’s breakfast skipping (full model, Table 3). Independent associations with lunch and dinner skipping were found for paternal educational level, and paternal educational level and household income, respectively (Tables 4 and 5).

**Table 3:** Associations of family socioeconomic position and ethnic background with breakfast skipping at age 6 years (n = 4687).

	Crude model	Basic model*	Full model**
	OR (95% CI)	OR (95% CI)	OR (95% CI)
Maternal educational level			
High (ref)	1.00	1.00	1.00
Mid-high	**2.21 (1.38,3.54)**	**1.70 (1.05,2.74)**	1.46 (0.88,2.43)
Mid-low	**3.82 (2.48,5.88)**	**1.97 (1.24,3.13)**	1.43 (0.85,2.42)
Low	**8.25 (5.28,12.90)**	**3.28 (2.01,5.35)**	**2.21 (1.24,3.94)**
Paternal educational level			
High (ref)	1.00	1.00	1.00
Mid-high	1.16 (0.73,1.85)	0.91 (0.57,1.45)	0.68 (0.42,1.11)
Mid-low	**2.50 (1.64,3.77)**	1.43 (0.93,2.19)	0.88 (0.54,1.42)
Low	**4.38 (2.96,6.47)**	**1.89 (1.23,2.91)**	1.02 (0.62,1.67)
Maternal employment status			
Paid job (ref)	1.00	1.00	1.00
No paid job	**2.14 (1.65,2.75)**	1.11 (0.82,1.50)	0.77 (0.55,1.09)
Paternal employment status			
Paid job (ref)	1.00	1.00	1.00
No paid job	**2.76 (1.81,4.19)**	1.45 (0.92,2.28)	1.17 (0.74,1.86)
Household income			
>€3200 (ref)	1.00	1.00	1.00
€2000-<€3200	**3.26 (2.29,4.64)**	**2.26 (1.55,3.29)**	**1.96 (1.27,3.04)**
<€2000	**6.70 (4.77,9.39)**	**3.02 (2.03,4.48)**	**2.43 (1.40,4.22)**
Family composition			
Two parents (ref)	1.00	1.00	1.00
Single parent	**1.99 (1.50,2.63)**	**1.57 (1.14,2.14)**	1.09 (0.76,1.57)
	Crude model	Basic model***	Full model****
	OR (95% CI)	OR (95% CI)	OR (95% CI)
Ethnic background			
Native Dutch (ref)	1.00	1.00	1.00
Surinamese-Creole	**5.37 (3.28,8.80)**	**5.29 (3.22,8.69)**	**3.36 (1.98,5.72)**
Surinamese-Hindustani	**4.62 (2.77,7.69)**	**4.55 (2.73,7.58)**	**2.95 (1.72,5.04)**
Dutch Antillean	**4.71 (2.65,8.40)**	**4.44 (2.48,7.97)**	**2.79 (1.50,5.17)**
Cape Verdean	**3.53 (2.09,5.98)**	**3.39 (2.00,5.76)**	**1.78 (1.01,3.14)**
Turkish	**7.30 (5.32,10.01)**	**7.27 (5.27,10.01)**	**4.27 (2.94,6.22)**
Moroccan	**7.21 (4.97,10.46)**	**7.10 (4.87,10.36)**	**4.03 (2.59,6.28)**

Table is based on imputed dataset. Bold print indicates statistical significance. Values represent odds ratios and 95% confidence intervals derived from (multiple) logistic regression analyses.

* Adjusted for child’s sex, child’s age, and ethnic background

** Additionally adjusted for all SEP indicators

*** Adjusted for child’s sex and child’s age

**** Additionally adjusted for all SEP indicators

**Table 4:** Associations of family socioeconomic position and ethnic background with lunch skipping at age 6 years (n = 4593).

	Crude model	Basic model*	Full model**
	OR (95% CI)	OR (95% CI)	OR (95% CI)
Maternal educational level			
High (ref)	1.00	1.00	1.00
Mid-high	1.29 (0.97,1.73)	1.20 (0.90,1.61)	1.08 (0.79,1.48)
Mid-low	**1.67 (1.28,2.19)**	1.31 (0.98,1.75)	1.02 (0.72,1.44)
Low	**2.72 (2.01,3.67)**	**1.80 (1.29,2.51)**	1.28 (0.84,1.95)
Paternal educational level			
High (ref)	1.00	1.00	1.00
Mid-high	1.08 (0.79,1.47)	1.00 (0.73,1.37)	0.96 (0.68,1.34)
Mid-low	**1.84 (1.37,2.47)**	**1.56 (1.15,2.12)**	1.42 (0.99,2.03)
Low	**2.43 (1.84,3.20)**	**1.80 (1.33,2.44)**	**1.53 (1.06,2.20)**
Maternal employment status			
Paid job (ref)	1.00	1.00	1.00
No paid job	**1.52 (1.23,1.89)**	0.90 (0.71,1.15)	0.97 (0.73,1.28)
Paternal employment status			
Paid job (ref)	1.00	1.00	1.00
No paid job	1.50 (0.98,2.28)	0.92 (0.59,1.46)	0.93 (0.57,1.51)
Household income			
>€3200 (ref)	1.00	1.00	1.00
€2000-<€3200	**1.47 (1.14,1.90)**	1.28 (0.98,1.67)	1.06 (0.79,1.42)
<€2000	**2.31 (1.84,2.90)**	**1.64 (1.24,2.15)**	1.23 (0.84,1.79)
Family composition			
Two parents (ref)	1.00	1.00	1.00
Single parent	**1.57 (1.23,2.00)**	**1.42 (1.09,1.84)**	1.17 (0.85,1.61)
	Crude model	Basic model***	Full model****
	OR (95% CI)	OR (95% CI)	OR (95% CI)
Ethnic background			
Native Dutch (ref)	1.00	1.00	1.00
Surinamese-Creole	**1.82 (1.14,2.90)**	**1.73 (1.08,2.77)**	1.29 (0.79,2.11)
Surinamese-Hindustani	0.82 (0.47,1.57)	0.83 (0.46,1.52)	0.65 (0.35,1.21)
Dutch Antillean	1.66 (0.97,2.85)	1.51 (0.88,2.61)	1.11 (0.63,1.96)
Cape Verdean	**2.11 (1.40,3.18)**	**2.02 (1.34,3.05)**	1.37 (0.88,2.14)
Turkish	**3.12 (2.41,4.04)**	**2.98 (2.30,3.87)**	**2.27 (1.67,3.08)**
Moroccan	**1.79 (1.24,2.58)**	**1.68 (1.16,2.42)**	1.24 (0.82,1.88)

Table is based on imputed dataset. Bold print indicates statistical significance. Values represent odds ratios and 95% confidence intervals derived from (multiple) logistic regression analyses.

* Adjusted for child’s sex, child’s age, and ethnic background

** Additionally adjusted for all SEP indicators

*** Adjusted for child’s sex and child’s age

**** Additionally adjusted for all SEP indicators

**Table 5:** Associations of family socioeconomic position and ethnic background with dinner skipping at age 6 years (n = 4537).

	Crude model	Basic model*	Full model**
	OR (95% CI)	OR (95% CI)	OR (95% CI)
Maternal educational level			
High (ref)	1.00	1.00	1.00
Mid-high	1.58 (0.92,2.71)	1.27 (0.73,2.21)	1.26 (0.70,2.27)
Mid-low	**1.87 (1.12,3.13)**	1.18 (0.68,2.05)	0.98 (0.51,1.89)
Low	**3.26 (1.90,5.61)**	**1.86 (1.02,3.40)**	1.31 (0.63,2.71)
Paternal educational level			
High (ref)	1.00	1.00	1.00
Mid-high	0.54 (0.29,1.03)	**0.47 (0.25,0.89)**	**0.39 (0.20,0.76)**
Mid-low	1.42 (0.88,2.27)	0.96 (0.59,1.58)	0.72 (0.41,1.59)
Low	**2.19 (1.34,3.56)**	1.25 (0.72,2.17)	0.81 (0.42,1.59)
Maternal employment status			
Paid job (ref)	1.00	1.00	1.00
No paid job	**1.82 (1.28,2.60)**	0.74 (0.50,1.10)	1.01 (0.65,1.57)
Paternal employment status			
Paid job (ref)	1.00	1.00	1.00
No paid job	**3.12 (1.78,5.45)**	**2.09 (1.14,3.84)**	1.67 (0.86,3.24)
Household income			
>€3200 (ref)	1.00	1.00	1.00
€2000-<€3200	**1.89 (1.16,3.06)**	1.57 (0.95,2.59)	1.70 (0.95,3.04)
<€2000	**3.75 (2.48,5.68)**	**2.42 (1.48,3.96)**	**2.44 (1.22,4.91)**
Family composition			
Two parents (ref)	1.00	1.00	1.00
Single parent	**1.79 (1.16,2.61)**	1.26 (0.81,1.98)	0.79 (0.47,1.33)
	Crude model	Basic model***	Full model****
	OR (95% CI)	OR (95% CI)	OR (95% CI)
Ethnic background			
Native Dutch (ref)	1.00	1.00	1.00
Surinamese-Creole	**3.82 (1.97,7.41)**	**3.85 (1.98,7.49)**	**2.91 (1.42,5.97)**
Surinamese-Hindustani	**2.58 (1.22,5.48)**	**2.59 (1.22,5.52)**	1.98 (0.90,4.37)
Dutch Antillean	**2.53 (1.07,5.96)**	**2.62 (1.10,6.21)**	1.90 (0.76,4.75)
Cape Verdean	**3.46 (1.84,6.54)**	**3.57 (1.88,6.77)**	**2.30 (1.13,4.68)**
Turkish	**2.78 (1.71,4.52)**	**2.76 (1.69,4.52)**	1.65 (0.93,2.94)
Moroccan	**3.64 (2.12,6.24)**	**3.63 (2.11,6.27)**	**1.93 (1.02,3.66)**

Table is based on imputed dataset. Bold print indicates statistical significance. Values represent odds ratios and 95% confidence intervals derived from (multiple) logistic regression analyses.

* Adjusted for child’s sex, child’s age, and ethnic background

** Additionally adjusted for all SEP indicators

*** Adjusted for child’s sex and child’s age

**** Additionally adjusted for all SEP indicators

After adjustment for child’s sex and age, ethnic minority children were more likely to skip breakfast than native Dutch children, with Turkish children showing the highest risk (basic model, Table 3). Turkish children were more likely to skip breakfast compared with Cape Verdean children but not compared with other ethnic minority children (data not shown). Following further adjustment for family SEP, all associations remained significant (full model, Table 3). Ethnic minority children, except for Surinamese-Hindustani and Dutch Antillean children, were more likely to skip lunch compared with native Dutch children (Table 4). Turkish children were significantly more likely to skip lunch than other ethnic minority children, except for Cape Verdean children (data not shown). Following adjustment for family SEP, only Turkish children were more likely to skip lunch compared with native Dutch children. With respect to dinner skipping, ethnic minority children were more likely to skip dinner than native Dutch children, with Surinamese-Creole children showing the highest risk (Table 5). Surinamese-Creole children did not significantly differ from other ethnic minority children (data not shown). After further adjustment for family SEP, the odds for skipping dinner remained increased for Surinamese-Creole, Cape Verdean, and Moroccan children. Analyses on weekly consumption of meals showed similar socioeconomic and ethnic inequalities (S2 and S3 Tables).

## Discussion

This study aimed to assess the prevalence and co-occurrence of breakfast, lunch, and dinner skipping among young school-aged children. Furthermore, the associations of family SEP and ethnic background with these meal skipping behaviors were investigated. The prevalence of meal skipping ranged from 3% (dinner) to 11% (lunch), and these meal skipping behaviors were moderately correlated. Meal skipping was more prevalent among low SEP children and ethnic minority children.

### Meal skipping behaviors

The prevalence of breakfast skipping is similar to that found in previous studies among 4- to 7-year-old children [6, 7, 30, 31]. Conversely, two studies conducted among Dutch children aged 4–6 years and 7–10 years reported lower prevalences of breakfast skipping (0% to 4%) [32, 33]. As these studies were performed in the ‘90s and increased rates of breakfast skipping have been observed [34], time of measurement may be an explanation for this discrepancy. An alternative explanation relates to the ethnic composition of the study population, with the current study encompassing more ethnic minority children (with higher rates of breakfast skipping) than these earlier studies. Research on lunch skipping and dinner skipping in young children is scarce; however, studies among older children (7- to 13-year-olds) show a higher prevalence of lunch skipping and dinner skipping [35–37]. Since meal skipping is known to increase with age throughout childhood and adolescence [33, 38], the age difference between studies may explain why we found a lower prevalence of lunch and dinner skipping.

Few studies have investigated the co-occurrence of meal skipping behaviors among young children and therefore direct comparison of the correlation coefficients found in the current study is precluded. However, in line with our findings, a study among 9- to 11-year-old Finnish children found similar proportions (i.e. 90%) of lunch and dinner consumers among regular breakfast consumers [35]. Under the assumption that determinants of breakfast, lunch, and dinner skipping largely overlap, larger correlation coefficients and more similar prevalences would have been expected. Based on the observation that breakfast consumption was more strongly associated with family SEP and ethnic background compared with lunch or dinner skipping, we speculate that the factors underlying breakfast skipping may be more structural than those underlying other meal skipping behaviors. For example, a shorter sleep duration or sleep quality, or a less organized household routine, could be associated with skipping breakfast rather than other main meals [18, 39]. Further research on the correlates of meal skipping, lunch and dinner skipping especially, in young children is merited.

### Socioeconomic inequalities in meal skipping behaviors

Our findings of socioeconomic inequalities in children’s meal skipping behaviors correspond to earlier studies showing higher levels of breakfast skipping among preschool children [6], school-aged children [9], and adolescents [13–17] from low SEP families. Our study adds to the limited evidence base on socioeconomic inequalities in lunch and dinner skipping behaviors by showing socioeconomic inequalities in both meal skipping behaviors. In a European-wide study among 10- to 12-year-old children, similar socioeconomic inequalities were found for dinner skipping but not for lunch skipping [17]. However, when analyses were presented for each country separately, results did show an (non-significant) increased risk of lunch skipping among low SEP children in the Netherlands. More research in this area is warranted.

The current study furthermore found independent associations between different SEP indicators and meal skipping behaviors. These independent associations are likely to represent different pathways connecting family SEP with meal skipping [26, 40, 41]. For example, in accordance with earlier studies [6, 41], we found maternal educational level and household income to be independently associated with breakfast skipping. Maternal educational level may exert its effects on meal skipping via knowledge and skills (e.g. parenting practices) acquired through education whereas household income is likely to represent financial resources available for food purchasing [26, 41]. Family composition has been consistently associated with breakfast skipping in previous research [18–20]. In the present study, the association between family composition and breakfast skipping attenuated after adjustment for other SEP indicators. These results suggest that the effects of family composition may not represent a separate pathway in the line of household organization or routines [19], but rather may be due to other socioeconomic characteristics of single parent families. The finding of an independent association between paternal educational level and dinner skipping, with children of mid-high educated fathers being less likely to skip dinner compared with children of high educated fathers, was an unexpected finding for which we currently have no explanation.

### Ethnic inequalities in meal skipping behaviors

The findings of ethnic inequalities in children’s breakfast skipping are in line with previous studies, most of which have been conducted in adolescent populations [6, 9, 12–17]. Moreover, two earlier Dutch studies among 4- to 10-year-old children also showed increased risks of breakfast skipping among Turkish and Moroccan children compared with native Dutch children [32, 33]. This study furthermore showed ethnic differences in lunch and dinner skipping. Contrary to our results, a European-wide study failed to find any ethnic disparities in lunch and dinner skipping when analyzing the total study population [17]. Due to pooling of data, existent ethnic inequalities within one country may have been obscured. Furthermore, for most of the countries, including the Netherlands, data on ethnic inequalities in lunch and dinner skipping were unavailable and therefore direct comparison of results is precluded.

Adjustment for family SEP attenuated results considerably for all meal skipping behaviors, indicating that the elevated risks of meal skipping among ethnic minority children are partly explained by adverse socioeconomic circumstances of the family. However, family SEP did not completely explain the associations of ethnic minority background and meal skipping behaviors, especially with respect to breakfast skipping. SEP independent effects of ethnic background on breakfast skipping have been reported by previous studies in both preschool children [6] and adolescents [12]. These findings indicate that, in addition to family SEP, cultural and/or social norms and values specific to these ethnic minority groups may further explain the increased risk of meal skipping among ethnic minority children. Barriers to improving the healthiness of children’s diet among ethnic minority groups may include (amongst others) language barriers, lack of control over feeding practices and food intake, lack of time to prepare food, dealing with child’s taste and preferences, and alternative priorities regarding a healthy development of children (e.g. children’s behavioral issues and concerns over safety and security) [42–44].

### Study strengths and limitations

The strengths of this study consist of the large and ethnically diverse study population and the availability of multiple indicators of family SEP. Several limitations should be considered when interpreting the results. First, nonresponse analyses showed that low SEP children and ethnic minority children more often had missing data on all three meal skipping behaviors compared with their counterparts. Bias due to selective participation may have occurred if the associations of family SEP and ethnic background with children’s meal skipping behaviors differ between participants and non-participants. However, as we have no data on non-participants this is difficult to ascertain. It may be assumed that low SEP and ethnic minority non-participants are worse off in terms of children’s health behaviors. Under this assumption, the social inequalities found in this study will be underestimations of the actual inequalities in the population. Second, potential information bias due to social desirable answering (i.e. over reporting of favorable behaviors and underreporting of unfavorable behaviors) may have been introduced by the use of parent-reported questionnaires. If meal skipping was underreported by parents of low SEP and ethnic minority children, observed associations underestimate true associations; however, this is difficult to ascertain. Moreover, at the age of 6 years, children in the Netherlands attend primary schools [45] and some children consume lunch at school. As a consequence, parents will be able to report on lunch being taken to school, but less so on lunch being actually consumed by their children. Also, we did not have information on types of food consumed during breakfast, lunch, and dinner and social inequalities in children’s consumption of food items during meals have been reported previously [1, 46]. Third, fully adjusted models were constructed to assess the independent effects of different SEP indicators and the SEP independent effects of ethnic background. Family SEP is a multidimensional construct and therefore difficult to capture completely [26, 27, 47]. Although we were able to control for a wide array of important socioeconomic indicators, residual confounding by unmeasured socioeconomic indicators such as wealth or neighborhood SEP cannot be ruled out [26, 27, 47]. Furthermore, family SEP may partly be on the causal pathway linking ethnic minority background with increased levels of meal skipping behaviors as impaired language proficiency and work floor discrimination may lead to lower levels of education, lesser job opportunities, and lower salaries for ethnic minority groups [48]. From this viewpoint, family SEP indicators may partly act as mediators rather than confounders. In a similar vein, part of the effects of socioeconomic indicators may be mediated through other socioeconomic indicators [49]. For example, the effects of educational level may be partly mediated through income as higher educated parents will be more likely to have a higher income. As a consequence, associations of ethnic background and indicators of family SEP, maternal educational level in particular, with meal skipping behaviors may have been underestimated when adjusting for (other) SEP variables.

## Conclusion

The prevalence of meal skipping behaviors among young ethnically diverse children range between 3% (dinner) and 11% (lunch). Breakfast skipping is moderately associated with lunch skipping and dinner skipping. Low SEP children and ethnic minority children are at an increased risk of breakfast, lunch, and dinner skipping compared with high SEP children and native Dutch children, respectively. Given these inequalities, interventions aimed to promote regular meal consumption, breakfast consumption in particular, should target children from low socioeconomic groups and ethnic minority children. More qualitative research to investigate the pathways underlying social inequalities in children’s meal skipping behaviors is warranted.

## Supporting Information

S1 Table Meal skipping behaviors at age 6 years. (DOCX)

S2 Table Number of meals consumed in the total population and according to family socioeconomic position and ethnic background (n = 4500). (DOCX)

S3 Table Associations of family socioeconomic position and ethnic background with number of meals consumed (n = 4500). (DOCX)
