# Supplementary material for: Social Inequalities in Young Children’s Meal Skipping Behaviors: The Generation R Study
Source: PLoS One. 2015 Jul 30;10(7):e0134487. doi: 10.1371/journal.pone.0134487 (PMC4520523; doi:10.1371/journal.pone.0134487)
Supplement: S1 Table — (DOCX) [file pone.0134487.s001.docx]

S1 Table. Meal skipping behaviors at age 6 years

| “On weekdays, how often does your child eat breakfast/ lunch/ dinner?” | Breakfast  (n=4687)  n (%) | Lunch  (n=4593)  n (%) | Dinner  (n=4573)  n (%) |
| --- | --- | --- | --- |
| 0 | 39 (0.8) | 24 (0.5) | 6 (0.1) |
| 1 | 12 (0.3) | 16 (0.3) | 8 (0.2) |
| 2 | 44 (0.9) | 26 (0.6) | 15 (0.3) |
| 3 | 70 (1.5) | 26 (0.6) | 19 (0.4) |
| 4 | 88 (1.9) | 71 (1.5) | 71 (1.6) |
| 5 | 4434 (94.6) | 4430 (96.5) | 4418 (97.4) |
| “In the weekends, how often does your child eat breakfast/ lunch/ dinner?” |  |  |  |
| 0 | 7 (0.1) | 43 (0.9) | 6 (0.2) |
| 1 | 73 (1.6) | 330 (7.2) | 43 (0.9) |
| 2 | 4607 (98.3) | 4220 (91.9) | 4488 (98.9) |
| Composite variable: weekly consumption of breakfast/ lunch/ dinner |  |  |  |
| 0* | 6 (0.1) | 8 (0.2) | 4 (0.1) |
| 1* | 2 (0.0) | 7 (0.2) | 1 (0.0) |
| 2* | 35 (0.7) | 17 (0.4) | 4 (0.1) |
| 3* | 14 (0.3) | 14 (0.3) | 9 (0.2) |
| 4* | 49 (1.0) | 31 (0.7) | 18 (0.4) |
| 5* | 66 (1.4) | 60 (1.3) | 21 (0.5) |
| 6* | 126 (2.7) | 349 (7.6) | 85 (1.9) |
| 7 | 4389 (93.6) | 4107 (89.4) | 4395 (96.9) |

Table is based on non-imputed dataset.

* Children with consumption < 7 days were considered breakfast, lunch, or dinner skippers
