# Supplementary material for: Social Inequalities in Young Children’s Meal Skipping Behaviors: The Generation R Study
Source: PLoS One. 2015 Jul 30;10(7):e0134487. doi: 10.1371/journal.pone.0134487 (PMC4520523; doi:10.1371/journal.pone.0134487)
Supplement: S2 Table — (DOCX) [file pone.0134487.s002.docx]

S2 Table. Number of meals consumed in the total population and according to family socioeconomic position and ethnic background (n=4500)

|  |  | Median | 90% Range | p-Value* |
| --- | --- | --- | --- | --- |
| Total population |  | 21 | 19-21 |  |
| Maternal educational level | High | 21 | 20-21 | <0.001 |
|  | Mid-high | 21 | 20-21 |  |
|  | Mid-low | 21 | 18-21 |  |
|  | Low | 21 | 16-21 |  |
| Paternal educational level | High | 21 | 20-21 | <0.001 |
|  | Mid-high | 21 | 20-21 |  |
|  | Mid-low | 21 | 18-21 |  |
|  | Low | 21 | 17-21 |  |
| Maternal employment status | Paid job | 21 | 19-21 | <0.001 |
|  | No paid job | 21 | 17-21 |  |
| Paternal employment status | Paid job | 21 | 19-21 | <0.001 |
|  | No paid job | 21 | 16-21 |  |
| Household income | >€3200 | 21 | 20-21 | <0.001 |
|  | €2000-<€3200 | 21 | 18-21 |  |
|  | <€2000 | 21 | 16-21 |  |
| Family composition | Two parents | 21 | 19-21 | <0.001 |
|  | Single parent | 21 | 17-21 |  |
| Ethnic background | Native Dutch | 21 | 20-21 | <0.001 |
|  | Surinamese-Creole | 21 | 17-21 |  |
|  | Surinamese-Hindustani | 21 | 18-21 |  |
|  | Dutch Antillean | 21 | 16-21 |  |
|  | Cape Verdean | 21 | 16-21 |  |
|  | Turkish | 21 | 16-21 |  |
|  | Moroccan | 21 | 16-21 |  |

Table is based on non-imputed dataset.

* P-Values assessed by Kruskal-Wallis tests
