# Supplementary material for: Social Inequalities in Young Children’s Meal Skipping Behaviors: The Generation R Study
Source: PLoS One. 2015 Jul 30;10(7):e0134487. doi: 10.1371/journal.pone.0134487 (PMC4520523; doi:10.1371/journal.pone.0134487)
Supplement: S3 Table — (DOCX) [file pone.0134487.s003.docx]

S3 Table. Associations of family socioeconomic position and ethnic background with number of meals consumed (n=4500)

|  | Crude model  β (95% CI) | Basic model*  β (95% CI) | Full model**  β (95% CI) |
| --- | --- | --- | --- |
| Maternal educational level |  |  |  |
| High (ref) | 0.00 | 0.00 | 0.00 |
| Mid-high | **-0.10 (-0.20,-0.00)** | -0.04 (-0.14,0.06) | -0.02 (-0.12,0.08) |
| Mid-low | **-0.26 (-0.25,-0.16)** | -0.08 (-0.17,0.02) | 0.00 (-0.11,0.12) |
| Low | **-0.71 (-0.83,-0.59)** | **-0.41 (-0.54,-0.28)** | **-0.28 (-0.44,-0.13)** |
| Paternal educational level |  |  |  |
| High (ref) | 0.00 |  | 0.00 |
| Mid-high | 0.00 -0.10,0.11) | 0.06 (-0.04,0.16) | 0.09 (-0.02,0.20) |
| Mid-low | **-0.26 (-0.38,-0.15)** | **-0.11 (-0.23,-0.00)** | -0.03 (-0.15,0.09) |
| Low | **-0.49 (-0.63,-0.35)** | **-0.23 (-0.37,-0.09)** | -0.07 (-0.23,0.09) |
| Maternal employment status |  |  |  |
| Paid job (ref) | 0.00 | 0.00 | 0.00 |
| No paid job | **-0.30 (-0.39,-0.21)** | -0.09 (-0.18,0.01) | 0.03 (-0.08,0.13) |
| Paternal employment status |  |  |  |
| Paid job (ref) | 0.00 | 0.00 | 0.00 |
| No paid job | **-0.40 (-0.62,-0.18)** | -0.15 (-0.37,0.08) | -0.04 (-0.27,0.18) |
| Household income |  |  |  |
| >€3200 (ref) | 0.00 | 0.00 | 0.00 |
| €2000-<€3200 | **-0.21 (-0.30,-0.13)** | **-0.12 (-0.20,-0.03)** | -0.07 (-0.17,0.03) |
| <€2000 | **-0.59 (-0.68,-0.50)** | **-0.32 (-0.43,-0.22)** | **-0.22 (-0.37,-0.06)** |
| Family composition |  |  |  |
| Two parents (ref) | 0.00 | 0.00 | 0.00 |
| Single parent | **-0.29 (-0.40,-0.19)** | **-0.18 (-0.29,-0.07)** | -0.02 (-0.15,0.11) |
|  | Crude model  β (95% CI) | Basic model***  β (95% CI) | Full model****  β (95% CI) |
| Ethnic background |  |  |  |
| Native Dutch (ref) | 0.00 | 0.00 | 0.00 |
| Surinamese-Creole | **-0.43 (-0.63,-0.23)** | **-0.41 (-0.61,-0.21)** | **-0.29 (-0.49,-0.08)** |
| Surinamese-Hindustani | **-0.32 (-0.51,-0.13)** | **-0.30 (-0.49,-0.11)** | -0.19 (-0.38,0.01) |
| Dutch Antillean | **-0.57 (-0.79,-0.35)** | **-0.53 (-0.75,-0.30)** | **-0.38 (-0.61,-0.15)** |
| Cape Verdean | **-0.52 (-0.71,-0.34)** | **-0.50 (-0.69,-0.32)** | **-0.30 (-0.49,-0.10)** |
| Turkish | **-0.76 (-0.88,-0.64)** | **-0.74 (-0.86,-0.61)** | **-0.54 (-0.67,-0.40)** |
| Moroccan | **-0.62 (-0.78,-0.47)** | **-0.59 (-0.74,-0.44)** | **-0.37 (-0.54,-0.20)** |

Table is based on imputed dataset. Bold print indicates statistical significance. Values represent betas and 95% confidence intervals derived from (multiple) linear regression analyses.

* Adjusted for child’s sex, child’s age, and ethnic background

** Additionally adjusted for all SEP indicators

*** Adjusted for child’s sex and child’s age

**** Additionally adjusted for all SEP indicators
